# Clinical examination, ultrasound assessment and aspiration of knee effusion in primary knee osteoarthritis patients

**DOI:** 10.1186/s13018-023-03891-6

**Published:** 2023-06-10

**Authors:** Noha Abdelhalim Elsawy, Aya Hanafy Ibrahiem, Gihan Abdellatif Younis, Marwa Ahmed Meheissen, Yousra Hisham Abdel-Fattah

**Affiliations:** 1 Department of Physical Medicine, Rheumatology and Rehabilitation, Faculty of Medicine, Alexandria University, Alexandria, Egypt; 2 Department of Medical Microbiology and Immunology, Faculty of Medicine, Alexandria University, Alexandria, Egypt

**Keywords:** Knee osteoarthritis, Effusion, Clinical examination, Ultrasound, Aspiration

## Abstract

**Background:**

To assess the diagnostic performance of clinical examination and ultrasound (US) assessment of knee effusion in primary knee osteoarthritis (KOA) patients. Furthermore, the success rate for effusion aspiration and the factors related to it were investigated.

**Methods:**

This cross-sectional study included patients diagnosed with primary KOA-induced knee effusion clinically or sonographically. The affected knee of each patient was subjected to clinical examination and US assessment using the ZAGAZIG effusion and synovitis ultrasonographic score. Patients with confirmed effusion and consented to aspiration were prepared for direct US-guided aspiration under complete aseptic techniques.

**Results:**

One hundred and nine knees were examined. During visual inspection, swelling was detected in 80.7% of knees and effusion was confirmed by US in 67.8% of knees. Visual inspection was the most sensitive at 90.54% while bulge sign was the most specific at 65.71%. Only 48 patients (61 knees) consented to aspiration procedure; 47.5% had grade III effusion, and 45.9% had grade III synovitis. Successful aspiration was achieved in 77% of knees. Two needle types were used; a 22 gauge / 3.5-inch spinal needle in 44 knees and an 18 gauge/ 1.5-inch needle in 17 knees, with a success rate of 90.9% and 41.2%, respectively. Aspirated amount of synovial fluid correlated positively with effusion grade (r_s=_0.455, *p* < 0.001) and negatively with synovitis grade on US (r_s_ = − 0.329, *p* = 0.01).

**Conclusions:**

The superiority of the US over clinical examination in detecting knee effusion suggests that US should be used routinely to confirm the presence of effusion. Long needles (spinal needle) may have a higher success rate of aspiration than shorter needles.

## Background

Knee joint effusion is the excessive aggregation of synovial fluid (SF) intra-articularly [1]. It can be caused by traumatic or non-traumatic factors such as knee osteoarthritis (KOA) [2]. The pathology of KOA involves the whole joint, including the synovial membrane [3]. In all grades of KOA, inflammation of the synovial membrane can be present, resulting in synovial membrane thickening and/or joint effusion [4]. Moderate to large effusions were reported in about 55% of patients with knee pain and radiographic osteoarthritis (OA) [5]. Knee effusion is associated with knee pain, decreased knee range of motion and quadriceps muscle weakness [5, 6]. Furthermore, knee effusion alters gait biomechanics [7].

Proper complete assessment of knee effusion requires clinical examination of the knee, imaging studies such as musculoskeletal ultrasound (US), aspiration, and analysis of SF. Clinical examination for knee effusion includes different clinical tests such as visual inspection, patellar tap test, balloon, and bulge sign [8–10]. There has been growing use of the point-of-care US for routine assessment of patients with KOA, a non-invasive imaging tool that is relatively inexpensive and does not require contrast [11, 12]. The definition of effusion in the suprapatellar pouch by US is ≥ 4 mm of fluid collection [13]. US demonstrated a sensitivity of 81.3% and a specificity of 100% for diagnosing knee effusion, making it comparable to magnetic resonance imaging (MRI) [12]. Meyer et al. [14] reported that US is more accurate than clinical examination and has higher sensitivity and specificity than MRI in detecting knee effusion [14].

Aspiration of knee effusion is a common procedure requiring adequate knowledge of joint anatomy to ensure aspiration success and avoid complications [15]. US-guided aspiration facilitates the detection and aspiration of effusions, even in small amounts [16–18]. Moreover, a randomized controlled trial by Sibbitt et al. [19] and a systematic review by Wu et al. [20] demonstrated that US-guided aspiration resulted in more successful aspiration of more significant amounts of SF with less pain during the procedure compared to landmark blinded aspiration. Several authors have reported that even with the utilization of US, aspiration of effusion could fail due to the similar appearance of different pathologies and other significant causes [21, 22]. To our knowledge, there is a lack of scientific literature about studies demonstrating the technical difficulties encountered during aspiration procedures, including the type of needle employed and the pathologies that impede aspiration.

This study aimed at assessing the diagnostic performance of clinical examination and US assessment of knee effusion in primary KOA patients. Also, the success rate for effusion aspiration and the factors related to it were investigated.

## Methods

### Studied patients

This cross-sectional study included patients diagnosed with primary KOA according to the 2010 evidence-based recommendations for the diagnosis of KOA by the European League Against Rheumatism [23]. The patients were recruited in the period from March 2021 to January 2022 from the outpatient clinic of the Physical Medicine, Rheumatology & Rehabilitation Department in Alexandria Main University Hospitals, Egypt.

#### Inclusion criteria

Adult patients ≥ 18 years of age diagnosed with primary KOA-induced knee effusion detected clinically or sonographically. The Inclusion criteria for aspiration of effusion [24] were as follows: 1. Patients with knee pain and swelling to decrease intraarticular pressure, 2. Before intraarticular injection of drugs. 3. Patients with an unclear diagnosis on US examination.

#### Exclusion criteria

Patients with secondary KOA, history of trauma or surgery to the knee, patients with known or suspected crystal-induced arthritis on clinical, laboratory or imaging studies and patients with a history of bleeding disorders.

All patients were subjected to the following:

1. Demographic data collection and calculation of body mass index (BMI) [25].
2. Complete history taking of the disease characteristics, focusing on previous or recurrent effusion and any previous intervention.
3. A Complete clinical examination of the knee [26] was done by an experienced rheumatologist with emphasis on knee pain assessment by visual analog scale (VAS); (0–10 cm) [27] with 0 referring to “no pain” and 10 to “maximum pain” and effusion examination including visible knee swelling, patellar tap test and bulge sign [9].
4. Plain X ray of the knee joint (postero anterior and lateral standing views) was done.
5. Musculoskeletal ultrasound examination using a 3–16 MHz linear array transducer (Samsung HS50, Korea) was done by an experienced rheumatologist to detect knee effusion and perform US-guided knee effusion aspiration. Longitudinal and transverse scanning of the suprapatellar and parapatellar pouches was done with the patient in a supine position with a knee flexion of 30 degrees. Additionally, scanning of the popliteal fossa was done for the detection of Baker’s cyst with the patient in a prone position [11]. The grading of synovitis, effusion, and Baker’s cyst was based on the ZAGAZIG ultrasonographic score [28]. The ZAGAZIG ultrasonographic score is a validated tool for ultrasonographic assessment of KOA which includes five domains: (*a) KOA severity (Grade (G) 0–4),* which depends on the shape of distal femoral osteophytes. (*b) Effusion (G 0–3)* in the form of abnormal anechoic or hypoechoic intra-articular material that is compressible and does not exhibit a Doppler signal. (*c) Synovitis (G 0–3)* in the form of abnormal hypoechoic or hyperechoic intra-articular tissue that is poorly compressible and may exhibit a Doppler signal. (*d) Pes anserine tendonitis/bursitis (G 0–2)*. (*e) Baker’s cyst (G 0–2)* in the form of a thin hypoechoic space delimited by echoic borders corresponding to the tissue-fluid interface anatomically present between the medial head of the gastrocnemius and the semimembranosus muscles [28]. However, only three domains were assessed in our study (effusion, synovitis, Baker’s cyst).
6. Preparation for effusion aspiration:
  - An informed consent was obtained.
  - Patient in a supine position with 15-20˚ knee flexion and a pillow under the knee. The patient was in a prone position with complete knee extension during aspiration from Baker’s cyst.
  - US-guided skin marking was used.
  - Complete aseptic technique was used.
7. Direct US-guided aspiration procedure:
  - Approach: Direct US-guided technique was used in all cases [29, 30], where the lateral approach was used in most cases, whereas the medial approach was used in cases where the lateral approach was inapplicable or difficult [31].
  - Equipment: Sterile single-use 20 ml luer lock syringe with an 18-gauge needle (1.5 inches) was successfully used in 17 knees, while it failed in other knees. Therefore, we used a sterile single use 20 ml luer lock syringe with a spinal needle (22-gauge/ 3.5 inches) [32] that resulted in successful aspiration in 44 knees.
  - One-needle multiple-syringe technique was used. The amount of aspirated SF was recorded, and routine culture and SF analysis were performed.

### Statistical analysis of the data

Data were fed into the computer, followed by statistical analysis using IBM-SPSS software package version 20.0. (Armonk, NY: IBM Corp). Descriptive statistics and means were used to describe the data. Categorical data were represented as numbers and percentages. The Chi-square test was applied to investigate the association between the categorical variables. Alternatively, Fisher’s Exact correction test was applied when the expected cell counts were less than 5. The Kolmogorov–Smirnov test for continuous data normality revealed that all data were abnormally distributed. The Spearman coefficient was used to determine the correlation between quantitative variables with an abnormal distribution. Kappa coefficient was used to examine the agreement between clinical examination and US. Kappa values of < 0 indicate no agreement, 0.01–0.20 slight agreement, 0.21–0.40 fair agreement, 0.41–0.60 moderate agreement, 0.61–0.80 substantial agreement and 0.81–1 almost perfect agreement. [33] For the diagnostic accuracy of clinical examination compared to US (gold standard) for detection of knee effusion, sensitivity, specificity, positive predictive value (PPV), and negative predictive value (NPV) were utilized. McNemar test was used to test the difference between clinical examination and US. The significance of the obtained results was judged at the 5% level.

## Results

**Fig. 1:**
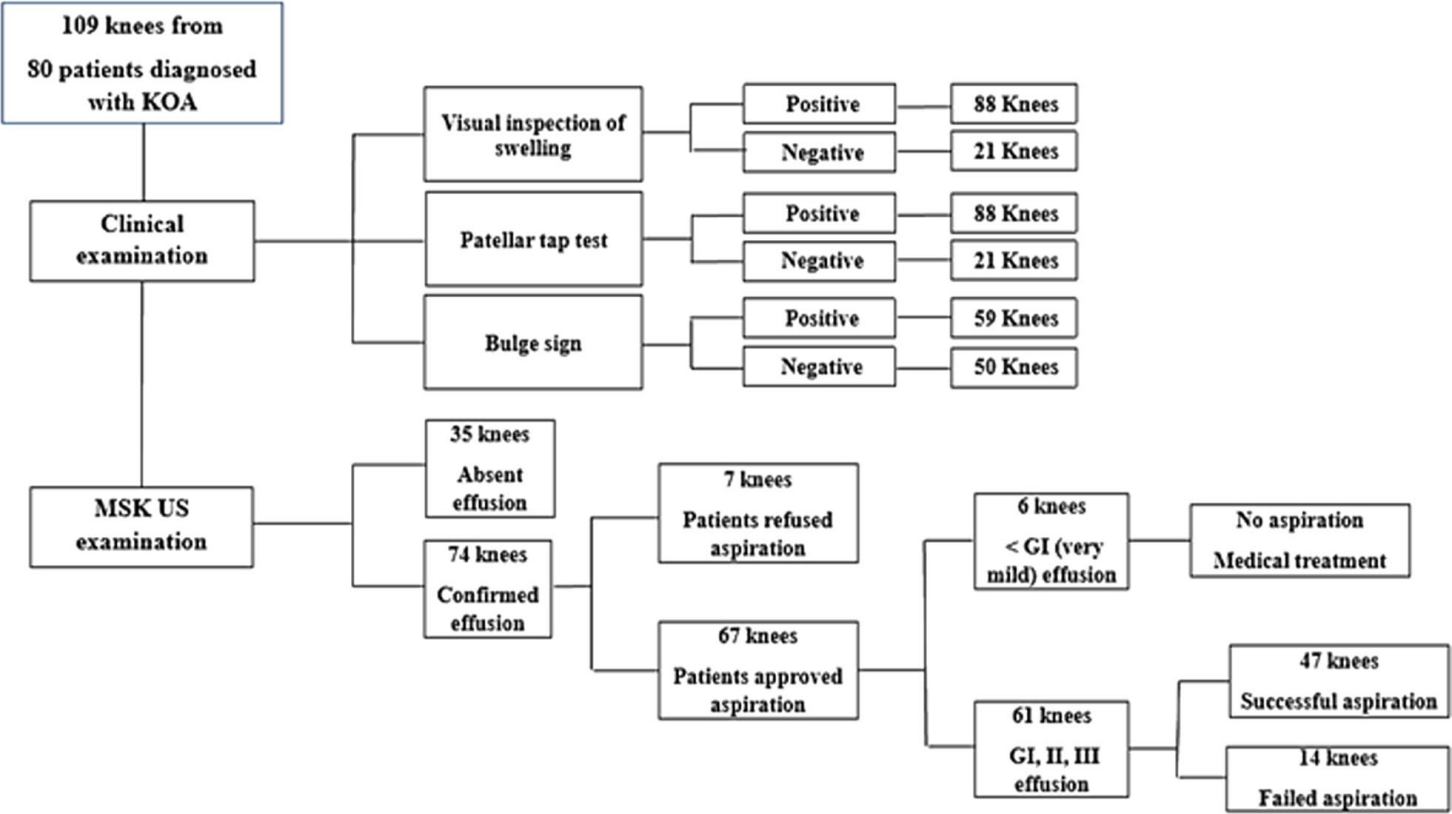
Flow chart for results

A total of 109 knees in 80 patients (16 males, 64 females) were examined clinically followed by US. Demographic characteristics, mean disease duration and mean VAS are outlined in Table 1. On clinical examination, 88/109 knees (80.7%) had positive swelling on inspection, 88/109 knees (80.7%) had positive patellar tap test, and 59/109 knees (54.12%) had positive bulge signs. On US scanning of the knees, effusion was confirmed in only 74/109 knees (67.8%) and negative in 35/109 knees (32.1%), as depicted in Fig. 1.

**Table 1:** Demographic characteristics, mean duration of the disease and mean VAS of the studied patients (n = 80 patients)

	Mean ± SD
Age (years)	59.4 ± 7.3
Weight (kg)	95 ± 13.4
Height (cm)	163.2 ± 5.2
BMI (kg/m^2^)	35.8 ± 5.7
Disease duration (years)	7.3 ± 4.2
VAS(cm)	7.2 ± 2.4

*SD* standard deviation, *Kg* kilogram, *cm* centimeter, *m*^*2*^ meter square, *VAS* visual analogue scale *BMI* body mass index

### Clinical examination results in relation to US findings

The results of different clinical examination tests and US for detection of knee effusion as well as the kappa coefficients, respective *p*-values and percentage of agreement are illustrated in Table 2. *p* value for kappa showed statistically significant difference between each of the clinical tests used and US. Furthermore, the visual inspection was the most sensitive at 90.54% while the bulge sign was the most specific at 65.71%. Diagnostic accuracy of clinical examination compared to US gold standard for detection of knee effusion is shown in Table 3. There was a statistically significant difference between the results of US versus the results of visual inspection of swelling (*p* = 0.008), patellar tap test (*p* = 0.024), and bulge sign (*p* = 0.024) in detecting knee effusion.

**Table 2:** Relation between ultrasound and clinical examination for knee effusion (n = 109 knees)

Clinical examination for knee effusion	Ultrasound			p ^κ^	Κ (LL–UL 95%C.I)	% agreement
	Negative (n = 35)	Positive (n = 74)	p			
Visual inspection of swelling						
Negative	13 (37.1%)	7 (9.5%)	< 0.001*	0.001*	0.312 (0.124–0.4999)	73.4%
Positive	22 (62.9%)	67 (90.5%)				
Patellar tap test						
Negative	11 (31.4%)	10 (13.5%)	0.027*	0.038*	0.200 (0.011–0.390)	68.8%
Positive	24 (68.6%)	64 (86.5%)				
Bulge sign						
Negative	23 (65.7%)	27 (36.5%)	0.004*	0.007*	0.263 (0.087–0.438)	64.2%
Positive	12 (34.3%)	47 (63.5%)				

κ kappa test, *CI* Confidence interval, *LL* Lower limit, *UL* Upper Limit

*p*: *p* value for comparing between Negative and Positive results of ultrasound and clinical examination (Chi square (*χ*^2^) test for qualitative variables)

p^κ^ refers to *p* value of Kappa coefficient

Kappa values of < 0 indicate no agreement, 0.01–0.20 slight agreement, 0.21–0.40 fair agreement, 0.41–0.60 moderate agreement, 0.61–0.80 substantial agreement and 0.81–1 almost perfect agreement

*Statistically significant at *p* ≤ 0.05

**Table 3:** Diagnostic accuracy of clinical examination compared to ultrasound (gold standard) for knee effusion detection (n = 109 knees)

	Visual inspection of swelling	Patellar tap test	Bulge sign
Sensitivity, (LL–UL 95%C.I)	90.54 (83.87–97.21)	86.49 (78.70–94.28)	63.51 (52.55–74.48)
Specificity, (LL–UL 95%C.I)	37.14 (21.13–53.15)	31.43 (16.05–46.81)	65.71 (49.99–81.44)
Positive predictive value, (LL–UL 95% CI)	75.28 (66.32–84.24)	72.73 (63.42–82.03)	79.66 (69.39–89.93)
Negative predictive value, (LL–UL 95% CI)	65.0 (44.10–85.90)	52.38 (31.02–73.74)	46.0 (32.19–59.81)
Accuracy, (LL–UL 95%C.I)	73.39 (65.10–81.69)	68.81 (60.11–77.50)	64.22 (55.22–73.22)
^McN^p	0.008*	0.024*	0.024*

*PPV* Positive predictive value, *NPV* Negative predictive value, *McN* McNemar test, *CI* Confidence interval, *LL* Lower limit, *UL* Upper Limit

*p*: *p* value for comparing between the studied groups

*Statistically significant at *p* ≤ 0.05

### Synovitis, effusion and Baker’s cyst grading

**Fig. 2:**
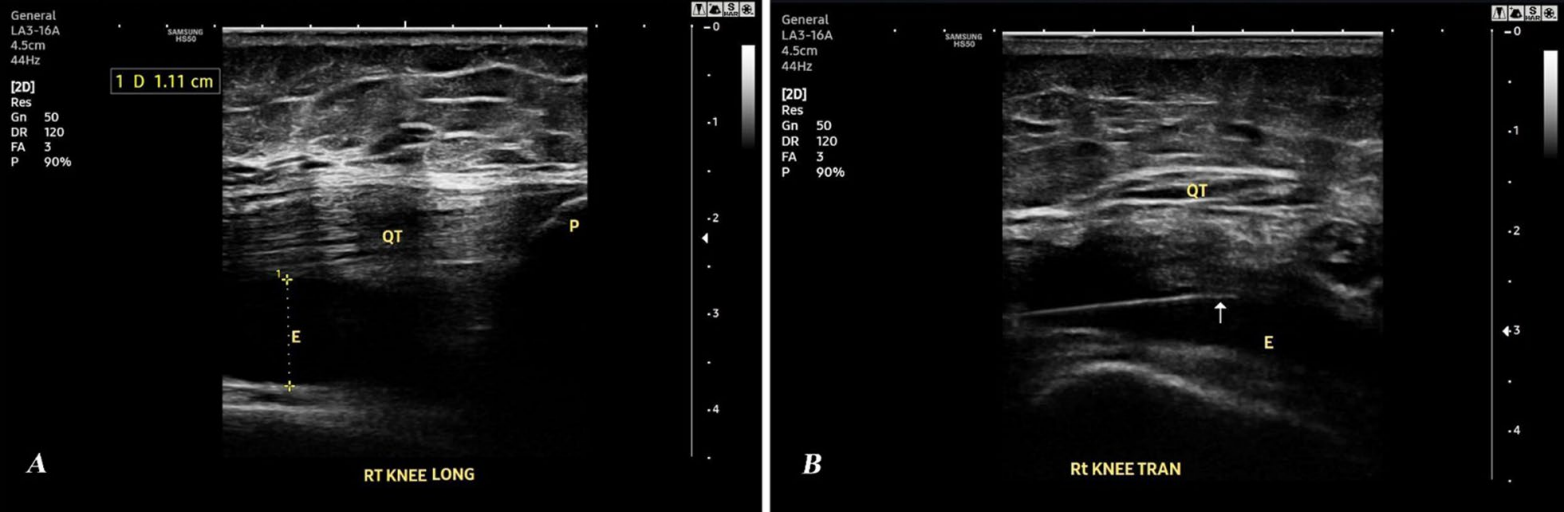
Ultrasound of the right knee of a 58-year-old knee osteoarthritis female patient showing effusion grade 3 and synovitis grade 2. **A** Longitudinal scanning. **B** Transverse scanning with 22-gauge spinal needle (white arrow). *QT* Quadriceps Tendon, *P* Patella, *E* Effusion

Upon obtaining the consent for effusion aspiration from the patients with confirmed effusion in US, only 48 patients (67 knees) approved aspiration and completed the study. Six knees (9%) had mild effusion (grade (G) I), with minimal knee pain, and were excluded from the aspiration procedure and prescribed medical treatment [24] (Fig. 1). The remaining 61 knees (91%) were assessed according to the ZAGAZIG ultrasonographic scale. The grading of synovitis, effusion and Baker’s cyst of these knees is shown in Table 4. An example of longitudinal and transverse US scanning of the right knee of a 58-year-old KOA female patient (effusion G 3 and synovitis G 2) who was successfully aspirated using a 22-gauge spinal needle is shown in Fig. 2.

**Table 4:** Synovitis, effusion & Baker’s cyst grading in knees with approved aspiration the according to ZAGAZIG ultrasonographic scale (n = 61 knees)

	n (%)
Synovitis grade	
I	12 (19.7%)
II	21 (34.4%)
III	28 (45.9%)
Effusion grade	
I	10 (16.4%)
II	22 (36.1%)
III	29 (47.5%)
Baker’s cyst grade	
0	37 (60.6%)
I	19 (31.1%)
II	5 (8.19%)

n: number, %: percentage

### Aspiration success or failure and the correlation between the amount of SF aspirated and the sonographic grading of synovitis and effusion in successfully aspirated knees

On aspiration, 47 knees (77%) had successful aspiration while 14 knees (23%) failed aspiration (Fig. 1). The synovitis and effusion grading for the 14 knees with failed aspiration was: 5 knees (35.71%) were GIII synovitis and GI effusion, 2 knees (14.29%) were GIII synovitis and GII effusion, 2 knees (14.29%) were GIII synovitis and GIII effusion, 2 knees (14.29%) were GII synovitis and GII effusion, 1 (7.14%) knee was GII synovitis and GIII effusion, 1 knee (7.14%) was GI synovitis and GII effusion and 1 knee (7.14%) was GI synovitis and GI effusion.

The median amount of SF aspirated was 12 (7–16.5) ml. There was a significant positive correlation between the amount of SF aspirated and the sonographic grading of effusion (r_s=_0.455, p < 0.001). Meanwhile, there was a significant negative correlation between the amount of SF aspirated and the sonographic grading of synovitis (r_s_ = −0.329, *p* = 0.01).

### The type of needle used

The spinal needle (22-gauge/3.5 inch) was used in 44 knees, of which 40 knees (90.9%) had successful aspiration. In contrast, a single-use 20 ml syringe with an 18-gauge (1.5 inch) needle was used in 17 knees, of which only 7 knees (41.2%) had successful aspiration. The other 10 knees (58.8%) with failed aspiration using the 18-gauge (1.5 inch) needle had effusion G I (mild) in 5 knees (50%), G II (moderate) in 4 knees (40%) and G III (severe) in 1 knee (10%). There was a statistically significant difference between the types of needles used regarding successful aspiration, with the spinal needle having a higher success rate (χ^2^ = 17.151, ^FE^*p* < 0.001).

### Culture and SF analysis results

The SF culture was negative in all cases. The SF aspirated from all knees was yellow in color with a clear aspect. The mean amount of proteins was 2.56 ± 0.82 g/dl, that of glucose was 94.80 ± 12.49 mg/dl, and that of LDH was 157.1 ± 33.04 U/L. The median amount of RBCs was 215 (102.5–325) cell/mm3. None of the patients had neutrophils or lymphocytes, and the results of SF analysis in all patients supported the diagnosis of non-inflammatory arthritis.

## Discussion

In the current study we analyzed the diagnostic performance of clinical examination versus US for detection of knee effusion in primary KOA patients. Furthermore, we investigated the success rate for effusion aspiration and its related factors.

### Detection of knee effusion by clinical examination versus US

In the current study, there was a great discrepancy in the detection of knee effusion between clinical examination and US. Our findings demonstrated that US was superior to all clinical examination tests utilized to confirm knee effusion. Similar findings were reported by Meyer et al. [14] and Ulasli et al. [34]. The agreement between US and both visual inspection and bulge sign tests in our study was fair, but statistically significant (κ = 0.312 *p* = 0.001, κ = 0.263 *p* = 0.007) respectively. Also, the agreement between US and patellar tap test was only slight but statistically significant (κ = 0.200 *p* = 0.038). Similarly, Ulasli et al. [34] found that the agreement of US results with second year resident clinical examination was slight (κ = 0.193, *p* = 0.007) and with senior resident clinical examination was fair (κ = 0.349, *p* < 0.001), but both were statistically significant.

Comparing different clinical tests against US (gold standard), our results indicated that clinical inspection had the highest sensitivity while bulge sign had the highest specificity of the clinical tests. Numerous studies demonstrated that different sensitivity and specificity of clinical tests were used to detect knee effusion versus US (gold standard) [8, 14, 34, 35]. Esen et al. [35] showed that inspection of a suprapatellar swelling in KOA patients had a sensitivity of 32.7% and specificity of 88.9%. Also, a systematic review by Meyer et al. [14] showed that clinical inspection had a sensitivity of 0.76 [0.59, 0.93], and physical examination had a sensitivity of 0.69 [0.59, 0.78] in detecting effusion versus US.

Several factors that might contribute to the variation in the detection of knee effusion between physical examination and the US were discussed, such as the size of clinically significant effusion, which is not clearly defined in the literature [14, 34]. Another factor was the number of years of experience required for accurate clinical examination [8, 14, 34, 35]. There has been a controversy over the effect of patients’ BMI on the diagnostic accuracy of clinical examination. In 1996, Roberts et al. [21] stated that obesity might negatively impact the clinical examination of joint effusion. Jaremko et al. [36] demonstrated that the sensitivity of the bulge sign increased when assessing patients with BMI less than 30 kg/m^2^. Conversely, Ulasli et al. [34] found no significant effect of BMI on the accuracy of clinical examination. Maricar et al. [8] concluded that there is still no standardized specific clinical test for effusion detection and eventually recommended imaging alongside clinical examination to confirm effusion.

Another important finding in our results is that when knee effusion results were positive in multiple clinical tests, it was negative in US, which helped avoid unnecessary arthrocentesis. This finding is comparable to Adhikari et al. [37] and Situ-LaCasse et al. [38], who found that the number of joints planned initially for aspiration decreased significantly after US assessment.

### Success rate for effusion aspiration

Our results showed that aspiration succeeded in 47 knees (77%), while it failed in 14 knees (23%) despite the fact that all knees were aspirated under US guidance. We attempted to outline some of the technical considerations that may aid in aspiration success in KOA patients, while decreasing failure rates.

Long needle length was one of the factors that contributed to aspiration Success. We utilized two different types of needles with variable lengths, and the results showed different success rates of aspiration. Successful aspiration was significantly higher with the spinal needle (22-gauge/ 3.5-inches) (90.9%) compared to the 18- gauge (1.5-inch) needle (41.2%). We attributed this result mainly to the length of the needle, as the shorter needle (1.5-inch) did not reach the site of effusion under US guidance in some cases. Our results showed that 6 (42.9%) knees with failed aspiration had mild effusion (GI), where the shorter length needle (1.5-inch) was used in 5 of them. Roberts et al. [21] discussed some of the main reasons for failed knee aspiration and mentioned that small amounts of effusion could be difficult to access. Therefore, we suggest that long-length needles may increase the success rate of effusion aspiration.

In addition, the majority of our patients were obese (mean BMI was 35.8 ± 5.7) and had increased subcutaneous fat thickness which made it difficult for the short needle (1.5-inch) to reach the effusion site. Therefore, the use of long, lengthened needles on obese patients could improve aspiration success. In this context, Hurdle et al. [32], successfully used a 22-gauge/ 3.5-inch spinal needle to aspirate SF from the knee of a morbidly obese patient under US guidance. Therefore, we hypothesized that the long, lengthened needles may have contributed to the success of aspiration in obese patients and those with mild knee effusion.

Extensive synovitis was a possible cause of aspiration failure in our study. Our results showed that 9 (64.3%) knees with failed aspiration had GIII (severe) synovitis, where 5 of them also had GI effusion. In some cases, the tip of the needle was plugged by the thickened synovium, hindering the flow of SF into the needle. This was supported by the negative correlation between the degree of synovitis and the amount of SF aspirated where the yielded amount of fluid aspirated was more in patients with lower degrees of synovitis. According to several studies, in patients with chronic synovitis, the synovium is less vascular with fibrotic pannus formation in which the fluid is sometimes replaced by fat (lipoma arborescens) [21, 22].

On the contrary, we found that intermittent compression using the US probe during aspiration increased the possibility of aspiration success and yielded more SF. This result is consistent with the study by Rolle et al. [39], who stated that manual or mechanical knee compression increased the rate of successful aspiration in KOA and rheumatoid arthritis patients.

The limitations of our study include the absence of two or more physicians with different years of experience who should have performed the clinical test for effusion (inter observer reliability), which would have increased the reliability of knee effusion clinical examination. Second, we find that the relatively high rate of aspiration failure in our study is an important limitation and all the possible causes of joint aspiration failure should be investigated in the future. Third, several patients refused effusion aspiration because they viewed it as an invasive, particularly during the COVID 19 pandemic.

## Conclusions

Knee effusion assessment in KOA patients is not as easy as it seems. It is essential to accurately assess knee effusion in order to determine the underlying pathology. The superiority of US over clinical examination in detecting knee effusion suggests that US should be used routinely to confirm the presence of effusion. Aspiration of knee effusion using long lengthened needles (spinal needle) guided by US may result in higher aspiration success rates than short lengthened needles.

## Data Availability

The datasets used and/or analyzed during the current study are available from the corresponding author on reasonable request.

## Abbreviations

BMI: Body mass index
G: Grade
KOA: Knee osteoarthritis
MRI: Magnetic resonance imaging
OA: Osteoarthritis
SF: Synovial Fluid
US: Ultrasound
VAS: Visual analog scale
